# Internal jugular phlebectasia as an incidental finding in cervical spine surgery

**DOI:** 10.4103/0019-5413.69324

**Published:** 2010

**Authors:** V Thulasiraman, TR Ramesh Pandian, S Cheralathan, S Ashok

**Affiliations:** Institute of Orthopedics and Traumatology, Madras Medical College and Government General Hospital, Chennai, India

**Keywords:** Cervical disc disease, cervical spine surgery, internal jugular phlebectasia

## Abstract

Idiopathic internal jugular phlebectasia, occurs either unilaterally or bilaterally affecting the internal jugular vein is a rare congenital variation often diagnosed during childhood. It usually presents with a benign swelling over the lateral side of neck on the affected side, seen on exertion. A-30-year old male was operated for anterior cervical dissectomy from right lateral approach and was diagnosed per-operatively as internal jugular phlebectasia. The surgery was abandoned at this stage on the advice of cardiothoracic surgeon to investigate the patient for the secondary etiological factors for internal jugular vein dilatation. The patient was reassured without any active intervention for the phlebectasia and cervical dissectomy was performed in the second surgery through the lateral approach from left side. This case is presented in view of rarity and suggested that during preoperative workup the nearby structures like carotid sheath should be evaluated by magnetic resonance imaging to avoid such per-operative surprises.

## INTRODUCTION

Idiopathic internal jugular phlebectasia is a rare congenital variation often diagnosed during childhood^1^. It occurs either unilaterally or bilaterally affecting the internal jugular vein.^2^ It usually presents with a benign swelling over the lateral side of neck on the affected side, seen on exertion and usually does not cause any other significant morbidity. Valsalva maneuver is the most useful clinical sign used to diagnose the dilatation of internal jugular vein.^2^ We report a case where internal jugular phlebectasia was diagnosed incidentally and per-operatively during the right lateral approach to cervical spine.

## CASE REPORT

A 30-year-old male, driver by occupation, presented with complaints of neck pain on and off for the past six months. He also gave history of pain radiating to the right upper limb, which aggravated on bending the neck forward. Occasional episodes of numbness over the thumb and adjacent areas were present.

**Figure 1:**
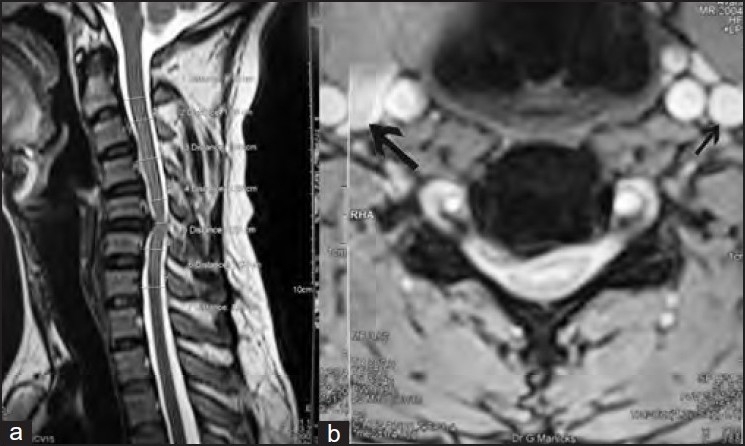
T2T1W sagittal MRI section of cervical spine (a) and horizontal cut section at C5C6 level (b) with dilated right internal jugular vein (long arrow) in comparison with left internal jugular vein of normal caliber (short arrow)

On clinical examination of neck, the patient was found to have no obvious external deformity. Minimal spinal tenderness was present over the C5 and C6 regions. Paraesthesia was present over the area of distribution of C6 nerve root, but there was no distal neurological deficit. The cervical X-ray showed obliteration of the normal cervical lordosis with no obvious vertebral lesion. The MRI of cervical spine [Figure 1] confirmed the presence of diffuse annular bulge with right posterior paracentral and foraminal disc protrusion seen at C5-C6 disc with impression on the dural sac and exiting nerve root on the right side with stenosis of right exiting neural foramina. There was no evidence of cord edema. All the routine haematological investigations were found to be within normal limits. Therefore final diagnosis of C5-C6 prolapse bulge with C6 radiculopathy was made. The patient had been on conservative management for the past six months with no satisfactory improvement in symptoms. Therefore the patient was planned for anterior discectomy and bone grafting and stabilization with cervical locking plate.

**Figure 2:**
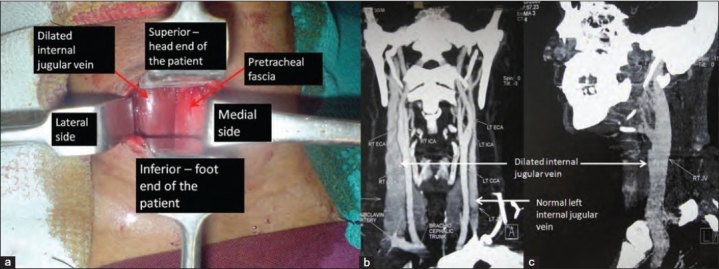
(a) Clinical photograph showing right sided anterior Smith Robinson approach of cervical spine with dilated internal jugular vein (arrow); CT venogram of the cervical spine, anteroposterior (b) and lateral (c) views shows dilated right internal jugular vein when compared to the normal left side

On the day of surgery under general anesthesia and through right lateral approach to the cervical spine, skin and the platysma were incised in the line of incision. While trying to retract the pretracheal fascia medially we noticed a bulging structure [Figure 2] protruding out, that felt soft like muscle but was compressible.

Stopping there we then retrospectively studied the pre-operative MRI film [Figure 1] of the cervical spine, which showed the right internal jugular vein to be enormously dilated in comparison to the left. This preoperative finding had been missed by the radiologist too. Being cautious not to damage the dilated internal jugular vein and to rule out intrathroracic causes^2^ such as anomalous reduplication of the jugular vein, pressure over the innominate vein causing jugular vein dilation, pressure of the scalene muscles that may cause secondary dilatation of internal jugular vein as also advised by the vascular surgeons, we abandoned the procedure without proceeding to discectomy and wound closure was done.

**Figure 3:**
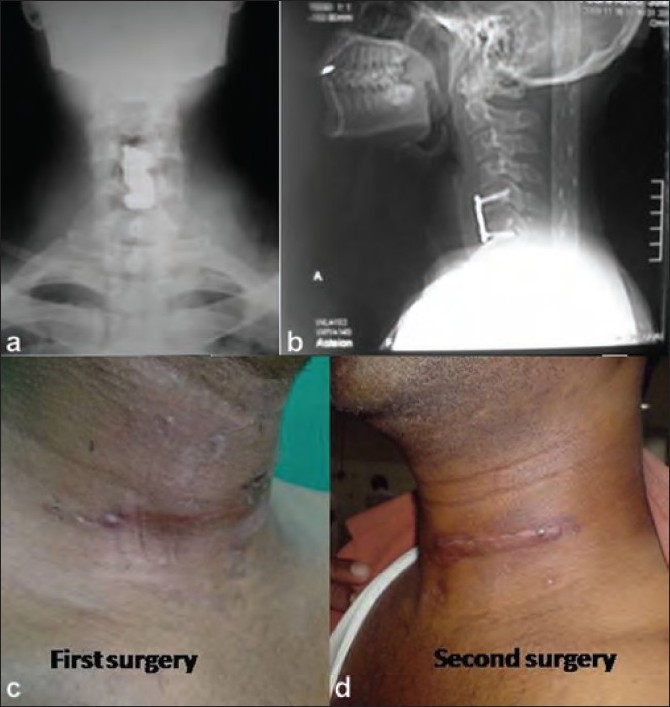
Post operative anteroposterior (a) and lateral (b) views of the cervical spine following anterior disectomy, bone grafting and stabilization with cervical locking plate. Clinical photographs, front (c) and side (d) views, showing healed scar

After the incidental per-operative finding, the patient was subjected to thorough investigations which included computerized tomography (CT) chest, CT abdomen, CT cervical venogram [Figure 2], duplex ultrasonogram (USG) of the cervical vascular structures. The possible conditions causing secondary internal jugular vein dilatation like intrathoracic space occupying lesions compressing over the great veins 2 were ruled out and hence the diagnosis of idiopathic internal jugular phlebectasia was made and the patient was taken up for the previosly planned surgery, using left sided approach to the cervical spine [Figure 3]. Postoperatively, the patient recovered well and in the third postoperative month, the patient had no neurological complaints or symptoms due to internal jugular vein phlebectasia.

## DISCUSSION

Internal jugular phlebectasia is a rare and benign condition occurring as a congenital variation. About 50 cases have been reported so far in literature;^3^ however, this is the first time it is being reported in cervical spine surgery. It is usually diagnosed during childhood.^1^,^4^,^5^ There have been cases that go unrecognized as in our case, which was discovered as an incidental per-operative finding. It is usually asymptomatic and the most useful clinical sign that can be found in a case on Internal jugular Phlebectasia is the Valsalva maneuver. Men are more commonly affected. The ratio varies from 2:1 to 3:2 and occurs more commonly on the right side, like the case presented here; the ratio being 5.8:1.^3^,^6^

The possible differential diagnosis for internal jugular vein phlebectasia are laryngocele (most common), arteriovenous malformations, cavernous hemangioma, branchial cyst and cystic hygroma.^1^,^5^,^7^ Internal jugular vein phlebectasia is not known to progress rapidly and there have been no instances of spontaneous rupture of the swelling or other serious complications. Though some authors advise surgical management for phlebectasia which is usually ligation and resection of the dilated segment of vein,^4^,^7^,^8^ it usually does not require any active intervention.^1^ Therefore, most of the authors advice only reassurance to the patients owing to the benign nature of phlebectasia.^1^,^8^

During evaluation of cervical spine disease with MRI it is necessary to see the disc and its implication but with the same interest one should also see nearby structures like carotid sheath to avoid such per-operative surprises.
